# Olfaction and muscle strength in older adults: a longitudinal study

**DOI:** 10.1093/gerona/glag125

**Published:** 2026-05-11

**Authors:** Rui Liu, Honglei Chen, Chenxi Li, Anna Kucharska-Newton, Eleanor M Simonsick, Yaqun Yuan

**Affiliations:** Department of Health Sciences, College of Health Professions, Sacred Heart University, Fairfield, Connecticut, United States; Department of Epidemiology and Biostatistics, College of Human Medicine, Michigan State University, East Lansing, Michigan, United States; Department of Epidemiology and Biostatistics, College of Human Medicine, Michigan State University, East Lansing, Michigan, United States; Department of Epidemiology, University of North Carolina at Chapel Hill, Chapel Hill, North Carolina, United States; Translational Gerontology Branch, Intramural Research Program of the National Institute on Aging, National Institutes of Health, Baltimore, Maryland, United States; Department of Epidemiology and Biostatistics, College of Human Medicine, Michigan State University, East Lansing, Michigan, United States; (Medical Sciences Section)

**Keywords:** Epidemiology, Olfactory impairment, Muscle strength, Aging, Longitudinal study

## Abstract

**Background:**

Olfactory impairment is common in older adults and has been linked to weight loss and functional decline. This study aimed to examine whether olfactory impairment is associated with an accelerated decline in muscle strength among older adults.

**Methods:**

We analyzed data from 2348 participants (aged 71-82 years; 48.0% men; 37.5% Black) in the Health, Aging, and Body Composition Study. Olfactory function was assessed using the Brief Smell Identification Test at the Year 3 clinical visit (1999-2000) and categorized as good (scores 11-12), moderate (9-10), hyposmia (7-8), or anosmia (0-6). Participants were followed for up to 7 years. Grip strength and quadriceps strength were measured at clinical visits in Years 4, 6, 8, and 10.

**Results:**

During follow-up, participants with anosmia experienced a faster decline in grip strength than those with good olfaction. The annual difference in decline was −0.19 kg/year (95% confidence interval [CI], −0.37 to −0.01) for men and −0.21 kg/year (95% CI, −0.37 to −0.05) for women. For quadriceps strength, men with anosmia had a greater annual decline (−1.26 Nm/year; 95% CI, −2.26 to −0.26), whereas no statistically significant association was observed in women.

**Conclusions:**

Anosmia in older adults is associated with accelerated decline in muscle strength. These findings suggest that olfactory impairment may serve as an early marker of neuromuscular aging. Further research is warranted to investigate the underlying mechanisms.

## Introduction

Loss of the sense of smell is common in older adults, affecting approximately 25% of individuals over 50 and more than 60% of those over 80.^1^ Beyond its established role as a prodromal marker of neurodegenerative diseases,^2–4^ olfactory impairment has also been independently associated with increased mortality.^5^ In recent years, emerging evidence has linked poor olfaction to aging-related functional decline, including mobility,^6^ physical functioning,^7^ and frailty.^8^ These findings suggest that olfactory impairment might be an early indicator of biological aging. Impaired olfaction may affect appetite and alter food preferences in older adults,^9^^,^^10^ leading to poor diet quality^9^ and loss of lean mass,^11^ which may in turn contribute to declines in muscle strength, a hallmark of aging. Maintaining muscle strength is critical for preserving mobility, independence, and overall health.^12^ Despite the biological plausibility, to our knowledge, only a few cross-sectional studies have examined this association,^13–16^ with some reporting a positive association between poor olfaction and lower grip strength,^14^^,^^16^ while others found null association.^13^^,^^15^ We hereby conducted the first longitudinal analysis to examine olfaction in relation to changes in muscle strength over 7 years in a large, biracial, community-based cohort of older adults.

## Methods

### Study population, olfaction, and muscle strength assessment

The Health, Aging, and Body Composition (Health ABC) Study is a prospective cohort of 3075 community-dwelling older adults (aged 70-79 years; 48.4% men; 41.6% Black), enrolled between 1997 and 1998 from Pittsburgh, Pennsylvania, and Memphis, Tennessee. The study was designed to investigate how changes in body composition affect health and function in aging. All participants were free of mobility limitations at enrollment and were followed through annual or biennial clinic visits and semiannual or quarterly telephone interviews. Detailed information on the study design has been published previously.^17^

Olfactory function was assessed at the Year 3 clinic visit (1999-2000; serving as the analytical baseline for this study) using the 12-item Brief Smell Identification Test (B-SIT), a validated screening tool widely used in epidemiological studies.^18^^,^^19^ Participants scratched and sniffed 12 common odorants and selected the correct answer from 4 choices. Scores ranged from 0 to 12, with lower scores indicating poorer olfactory function. Consistent with prior studies,^6^ we defined anosmia as a B-SIT score of 0-6, hyposmia as 7-8, moderate olfaction as 9-10, and good olfaction as 11-12.

Muscle strength was assessed through grip and quadriceps strength measurements during clinical visits at Years 4, 6, 8, and 10. Grip strength in kilograms (kg) was measured using a hand-held dynamometer (Jamar, Sammons Preston Rolyan, Bolingbrook, IL). Participants were seated in a chair with the tested arm resting on a table and the elbow flexed at approximately a 90° angle. The dynamometer was calibrated for each participant according to hand size. Participants completed 2 trials per hand and the maximum value of 4 trials was used in the analysis.^20^ Quadriceps strength was assessed using a Kin-Com 125 AP isokinetic dynamometer (Chattanooga, TN). Participants were seated with the lateral femoral epicondyle aligned with the dynamometer axis and performed concentric knee extensions at 60°/s through a range of 90°-30° of knee flexion, with gravity correction applied. Testing was performed on the right leg unless pain or joint replacement precluded it, in which case the left leg was assessed. After 2 submaximal practice trials at ∼50% effort, participants completed 3-6 maximal efforts, and torque curves were overlaid until 3 consistent curves were obtained. The final score was the mean peak torque (Nm) across those 3 maximal trials.^21^

Among the 2537 participants who completed the B-SIT at Year 3, those with missing covariates (*n* = 31) or without any grip or quadriceps strength measurements were excluded. The final analytic sample included 2348 participants for grip strength and 2201 for quadriceps strength (Figure S1). Compared to included participants, those excluded were slightly older and generally less healthy (Table S1).

### Statistical analysis

We used a joint modeling approach^22^ to examine the associations between olfaction and changes in muscle strength over time. This approach combines a survival and a longitudinal submodel to account for potential biases arising from death or loss to follow-up. Specifically, we fitted a cause-specific Cox hazard model for the competing risks of death and dropout, and a linear mixed-effect model for each muscle strength measure, with subject-specific random intercepts and slopes. Participants were followed from baseline until death, last eligible visit, or the end of the follow-up, whichever occurred first. We tested the linearity of strength trajectories by including a quadratic time term in the mixed-effects model. The term was not statistically significant for either grip or quadriceps strength, supporting the assumption of linear decline.

Covariates were selected based on previous literature,^6^ including age, race, study site, education, smoking, alcohol drinking, brisk walking (as a surrogate for physical activity), body mass index (BMI), self-rated health, and major chronic diseases. Chronic diseases were defined using established criteria, including adjudicated diagnoses of diabetes,^23^ cardiovascular diseases,^24^ kidney disease,^25^ dementia,^26^ Parkinson’s disease,^4^ and depressive symptoms.^27^ Covariates were from the Year 3 visit, except for study site, race, education, and alcohol drinking, which were collected at Year 1.

Participants with good olfaction were considered the reference group. All analyses were stratified by sex, given the well-documented sex difference in muscle strength^28^ and similar patterns were observed in our data (Figure S2). We conducted sensitivity analyses by excluding participants with prevalent dementia or Parkinson’s disease at baseline due to their known associations with olfactory impairment. All analyses were performed using SAS, version 9.3 (SAS Institute, Inc.), and the JM package (version 1.4-5) in R.^22^ Statistical significance was inferred when 95% confidence intervals (CIs) excluded the null value.

## Results

At baseline (Year 3 clinic visit, mean age 75.6 ± 2.8 years, range 71-82), participants with anosmia or hyposmia were generally older and more likely to be male, Black, or ever smokers, and to have lower educational attainment than those with good olfaction. They were also more likely to report fair-to-poor health status and to have prevalent diabetes, chronic kidney disease, dementia, Parkinson’s disease, and depressive symptoms (Table S2).

### Olfaction and grip strength

**Figure 1:**
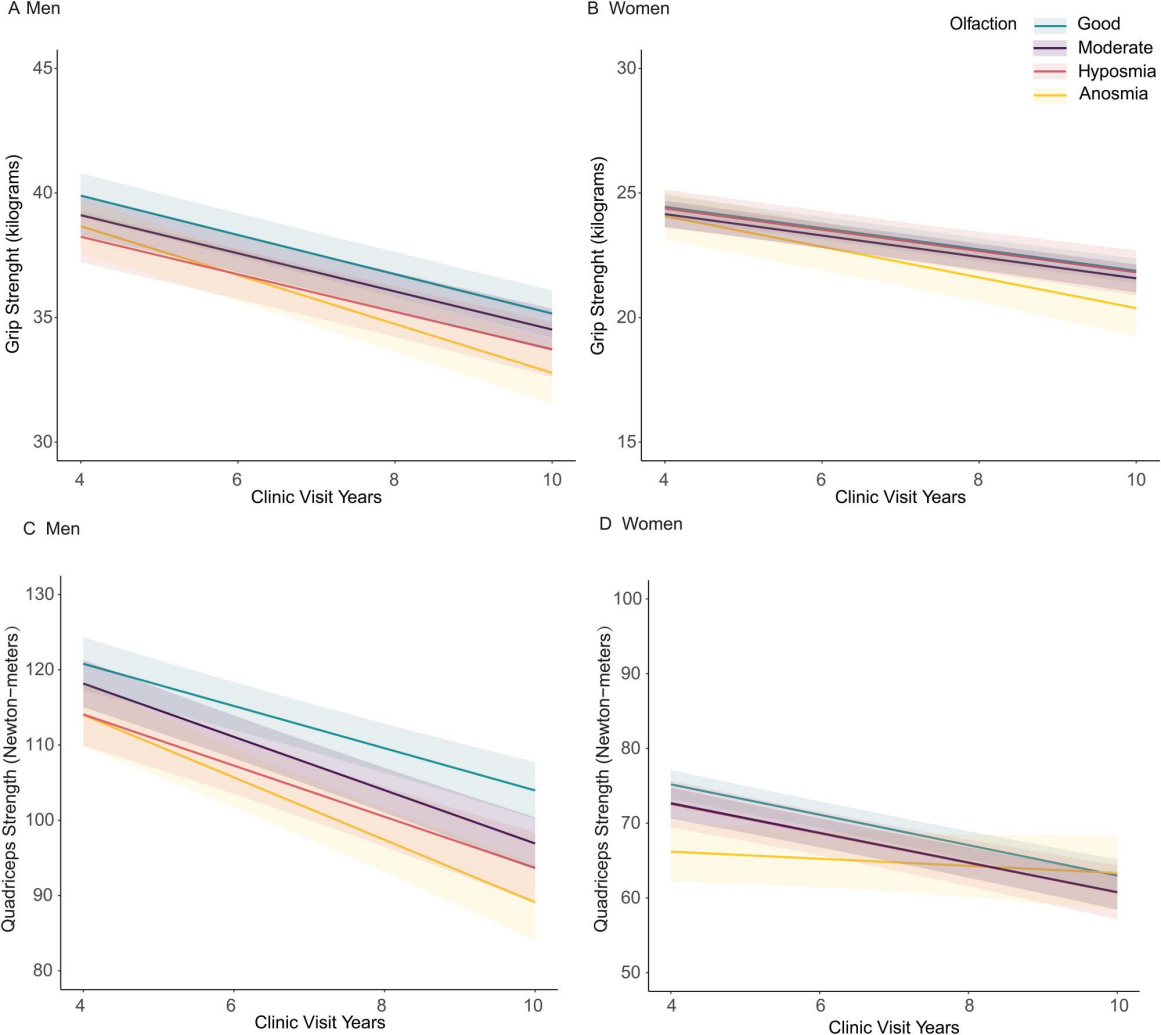
Unadjusted mean trajectories of grip strength and quadriceps strength, by sex and baseline olfaction. Loss of follow-up and death are accounted for by joint model analyses. (A) Men, grip strength; (B) Women, grip strength; (C) Men, quadriceps strength; (D) Women, quadriceps strength.

Compared with older adults with good olfaction, those with anosmia, hyposmia, and moderate olfaction had lower estimated grip strength at baseline, although differences were not statistically significant (Table 1). During follow-up, grip strength declined across all olfaction groups (Figure 1A and B). Among those with good olfaction, the rate of decline was −0.79 kg/year (95% CI, −0.89 to −0.68) for men and −0.42 kg/year (95% CI, −0.48 to −0.36) for women. Compared with this reference group, participants with anosmia experienced a faster decline in grip strength, with an annual difference of −0.19 kg/year (95% CI, −0.37 to −0.01) for men and −0.21 kg/year (95% CI, −0.37 to −0.05) for women (Table 1). No statistically significant differences were observed for participants with hyposmia or moderate olfaction. Results were similar in sensitivity analyses excluding participants with prevalent Parkinson’s disease and/or dementia (Table S3).

**Table 1:** Olfaction in relation to grip strength at baseline and annual declines in the health ABC study.

	Between-group difference with good olfaction as the reference (95% CI)^a^	
	Men	Women
	*N* = 1127	*N* = 1221
**Olfaction and baseline grip strength (kg)^b^**		
Anosmia	−0.62 (−1.98 to 0.75)	−0.27 (−1.36 to 0.79)
Hyposmia	−1.41 (−2.65 to −0.12)	−0.07 (−0.93 to 0.83)
Moderate	−0.86 (−1.93 to 0.18)	−0.48 (−1.17 to 0.22)
Good	Ref.	Ref.
Annual decline in participants with good olfaction (kg/year)	−0.79 (−0.89 to −0.68)	−0.42 (−0.48 to −0.36)
**Olfaction and annual decline in grip strength (kg)**		
Anosmia	−0.19 (−0.37 to −0.01)	−0.21 (−0.37 to −0.05)
Hyposmia	0.04 (−0.13 to 0.20)	−0.01 (−0.14 to 0.10)
Moderate	0.03 (−0.11 to 0.17)	0.00 (−0.09 to 0.08)
Good	Ref.	Ref.

Abbreviation: Ref., reference.

a Estimated values were obtained from joint models, adjusting for age, race, clinical site, education, smoking, alcohol drinking, brisk walking, BMI, general health status, cardiovascular diseases, chronic kidney diseases, depressive syndromes, and dementia and Parkinson’s disease, and the loss of follow-up and death.

b Estimated between-group difference in grip strength at baseline, with good olfaction as the reference.

### Olfaction and quadriceps strength

Among men, findings for quadriceps strength were consistent with those for grip strength.

Although the estimated cross-sectional differences were not statistically significant, men with anosmia had a faster decline in quadriceps strength (−1.26 Nm/year, 95% CI, −2.26 to −0.26) compared with those with good olfaction, who had a decline rate of −2.78 Nm/year (95% CI, −3.37 to −2.19; Figure 1C, Table 2). Among women, those with anosmia had a significant lower quadriceps strength (−7.65 Nm, 95% CI, −12.3 to −3.01) estimated at baseline than those with good olfaction. Over time, compared with women with good olfaction, who had a decline rate of −2.02 Nm/year (95% CI, −2.33 to −1.72), those with anosmia showed a slower rate of decline (1.49 Nm/year, 95% CI, 0.67-2.31; Figure 1D, Table 2). Sensitivity analyses obtained similar findings (Table S4).

**Table 2:** Olfaction in relation to quadriceps strength at baseline and annual declines in the health ABC study.

	Between-group difference with good olfaction as the reference (95% CI)^a^	
	Men	Women
	*N* = 1062	*N* = 1139
**Olfaction and baseline quadriceps strength (Nm)^b^**		
Anosmia	−1.85 (−7.81 to 4.11)	−7.65 (−12.3 to −3.01)
Hyposmia	−2.65 (−8.37 to 3.06)	−0.25 (−4.00 to 3.50)
Moderate	−1.02 (−5.95 to 3.91)	−1.68 (−4.53 to 1.17)
Good	Ref.	Ref.
Annual decline in participants with good olfaction (Nm/year)	−2.78 (−3.37 to −2.19)	−2.02 (−2.33 to −1.72)
**Olfaction and annual decline in quadriceps strength (Nm)**		
Anosmia	−1.26 (−2.26 to −0.26)	1.49 (0.67 to 2.31)
Hyposmia	−0.59 (−1.53 to 0.36)	0.03 (−0.59 to 0.65)
Moderate	−0.75 (−1.55 to 0.05)	0.07 (−0.38 to 0.52)
Good	Ref.	Ref.

Abbreviation: Ref., reference.

a Estimated values were obtained from joint models, adjusting for age, race, clinical site, education, smoking, alcohol drinking, brisk walking, BMI, general health status, cardiovascular diseases, chronic kidney diseases, depressive syndromes, and dementia and Parkinson’s disease, and the loss of follow-up and death.

b Estimated between-group difference in quadriceps strength at baseline, with good olfaction as the reference.

## Discussion

To our knowledge, this study provides one of the first longitudinal evidence that anosmia is associated with declines in muscle strength among older adults. This association was observed for both grip strength and quadriceps strength and was generally consistent across men and women. The findings remained robust after adjusting for multiple covariates and accounting for potential bias due to death or loss to follow-up.

Prior research on olfaction and muscle strength has been limited to cross-sectional studies with inconsistent results. A study of 141 community-dwelling older adults in Japan reported that olfactory impairment, defined as correctly identifying 7 or fewer odors on a 12-item test, was associated with low grip strength (<26 kg in men and <18 kg in women).^14^ In contrast, 2 other small studies, one in Japan (*n* = 130)^15^ and one in Australia (*n* = 957),^13^ reported no association. A larger cross-sectional analysis of 2861 U.S. older adults showed that better odor identification was associated with higher grip strength, but sex-specific associations were not examined.^16^

The Health ABC Study was uniquely designed to investigate changes in body composition and functional outcomes among initially well-functioning older adults. Its longitudinal design and repeated assessments of both upper- and lower-extremity strength enabled a comprehensive examination of the association between olfaction and muscle strength over time. The inclusion of sex-stratified analyses and the application of joint modeling to address attrition bias further enhance the robustness of our findings.

Muscle strength declines with age, with sharper reductions typically occurring after age 50.^29^ Grip strength is widely recognized as a proxy for overall muscle strength and has been strongly associated with adverse outcomes in older adults.^30^ In our study, participants with anosmia experienced an additional annual decline in grip strength of approximately 0.19-0.21 kg. Prior research has suggested that even modest changes in grip strength could be clinically meaningful. For example, in the Women’s Health and Aging Study II, Xue et al.^31^ reported that a decline in grip strength of just 0.07 kg/year (equivalent to 0.5 standard deviation) was associated with an increased risk of falls, slower walking speed, IADL disability, and frailty. These findings indicate that relatively small differences in the rate of strength decline can have meaningful functional consequences. Therefore, the additional annual loss observed among participants with anosmia in our study may accelerate functional deterioration over time.

To the best of our knowledge, no prior study has examined the association between olfaction and quadriceps strength. As a key indicator of lower-limb strength, quadriceps strength has been associated with independence in activities of daily living^32^ and an increased risk of falls.^33^ In our study, the pattern of quadriceps strength decline across olfaction groups in men was consistent with that observed for grip strength, suggesting that anosmia may be associated with deterioration in both upper- and lower-body muscle strength. Among women, however, those with anosmia had significantly lower baseline quadriceps strength yet a slower rate of decline compared with those with good olfaction. Although the explanation of this differential finding is uncertain, selection bias may partly account for it. As the isokinetic quadriceps test is more physically demanding, those with poorer lower-limb function at baseline (eg, women with anosmia in our sample) may have been less likely to complete the assessment over time, leaving those with relatively better lower-limb function in the analysis and showing a slower rate of decline. This possibility is supported by our prior finding that older adults with poor olfaction were less likely to complete the 400-m fast-walking test, a physically demanding task.^6^ However, future studies with larger samples and younger populations should further investigate this association.

The interpretation of this association remains uncertain and speculative, partly because of limited research on olfaction in older adults. While olfactory impairment may signify accelerated biological aging, it could also play an etiological role in the deterioration of muscle strength and physical function. Impaired sense of smell in older adults may reduce appetite and alter food preferences,^10^ potentially leading to inadequate protein and macronutrient intake,^9^ and further resulting in loss of muscle mass and strength. Supporting this possibility, our prior work found that poor olfaction was associated with greater loss of lean mass over time.^11^ Taken together, these findings suggest that assessing olfactory function may help identify older adults at risk and guide early strategies to preserve muscle and physical function.

This study has several limitations. First, participants were relatively older (71-82 years at the time of olfaction assessment), which may limit generalizability to younger populations. Second, olfaction was measured only once, precluding the evaluation of changes over time. Third, the B-SIT does not distinguish age-related decline from other causes, such as nasal surgery or chronic rhinosinusitis, although olfactory impairment at this age is presumed to be age-related. Fourth, excluded participants were slightly older and generally less healthy than those included. Fifth, we found that only anosmia, not hyposmia or moderate olfaction, was associated with faster decline in muscle strength, which is inconsistent with our prior work demonstrating a dose-response association between olfactory function and mobility.^6^ The explanation for this discrepancy remains unclear and warrants investigation in future studies with larger samples. Finally, as with all observational studies, residual confounding cannot be completely ruled out.

In conclusion, anosmia in older adults was associated with accelerated decline in muscle strength. Future studies should investigate the underlying mechanisms and their potential health implications in older adults.

## Supplementary Material

glag125_Supplementary_Data

## Data Availability

Dr. Yaqun Yuan had full access to all the data in the study and takes responsibility for the integrity of the data and the accuracy of the data analysis. Data used in the present analysis are available from the National Institute on Aging Health ABC study. To access the data, investigators should submit an analytic proposal for approval (https://healthabc.nia.nih.gov/analysis-proposals-publications).
