# Supplementary material for: Olfaction and muscle strength in older adults: a longitudinal study
Source: J Gerontol A Biol Sci Med Sci. 2026 May 11;81(7):glag125. doi: 10.1093/gerona/glag125 (PMC13242264; doi:10.1093/gerona/glag125)
Supplement: glag125_Supplementary_Data [file glag125_supplementary_data.docx]

**Olfaction and Muscle Strength in Older Adults: A Longitudinal Study**

Online Data Supplement

eTable 1. Baseline characteristics of participants who were included versus excluded from the analysis of quadriceps strength

eTable2. Baseline population characteristics by olfaction status (n=2348)

eTable 3. Olfaction and grip strength, excluding prevalent cases of dementia or Parkinson’s disease

eTable 4. Olfaction and quadriceps strength, excluding prevalent cases of dementia or Parkinson’s disease

eFigure 1. Flowchart of study participation

eFigure 2. Distribution of muscle strength, by clinic visit years and sex

| eTable 1. Baseline characteristics of participants who were included versus excluded from the analysis of quadriceps strength. | | | |
| --- | --- | --- | --- |
|  | Participants included in the analysis (n=2201) | Participants excluded (n=336) | *P-*Value |
| Mean age (SD), year | 75.5 ± 2.8 | 76.2 ± 2.8 | <.001 |
| Sex (%) |  |  |  |
| Men | 1062 (48.3) | 165 (49.1) | 0.77 |
| Women | 1139 (51.7) | 171 (50.9) |  |
| Race |  |  |  |
| White | 1386 (63.0) | 175 (52.1) | .001 |
| Black | 815 (37.0) | 161 (47.9) |  |
| Study site (%) |  |  |  |
| Memphis | 1065 (48.4) | 172 (51.2) | .34 |
| Pittsburgh | 1136 (51.6) | 164 (48.8) |  |
| Education (%) |  |  |  |
| Less than high school | 479 (21.8) | 93 (27.7) | .01 |
| High school | 725 (32.9) | 110 (32.7) |  |
| Above high school | 997 (45.3) | 126 (37.5) |  |
| Missing |  | 7 (2.1) |  |
| Smoking status (%) |  |  |  |
| Non-smoker | 1004 (45.6) | 133 (39.6) | .05 |
| Ever-smoker | 1197 (54.4) | 200 (59.5) |  |
| Missing |  | 3 (0.9) |  |
| Drinking alcohol (%) |  |  |  |
| Never | 616 (28.0) | 86 (25.6) | .545 |
| Ever Drinking | 1585 (72.0) | 240 (71.4) |  |
| Missing |  | 10 (3.0) |  |
| Brisk walking (%) |  |  |  |
| <90 min/week | 1966 (89.3) | 317 (94.4) | .01 |
| ≥90 min/week | 235 (10.7) | 18 (5.3) |  |
| Missing |  | 1 (0.3) |  |
| Body mass index (kg/m^2^, %) |  |  |  |
| <25 | 739 (33.6) | 115 (34.2) | .02 |
| 25-30 | 947 (43.0) | 120 (35.7) |  |
| >30 | 515 (23.4) | 99 (29.5) |  |
| Missing |  | 2 (0.6) |  |
| General health status (%) |  |  |  |
| Excellent to good | 1851 (84.1) | 233 (69.4) | <.001 |
| Fair to poor | 350 (15.9) | 100 (29.8) |  |
| Missing |  | 3 (0.8) |  |
| Diabetes (%) |  |  |  |
| No | 1709 (77.6) | 233 (69.3) | <.001 |
| Yes | 492 (22.4) | 103 (30.7) |  |
| Cardiovascular diseases (%) |  |  |  |
| No | 1616 (73.4) | 187 (55.7) | <.001 |
| Yes | 585 (26.6) | 149 (44.3) |  |
| Chronic kidney disease (%) |  |  |  |
| No | 1719 (78.1) | 205 (61.0) | <.001 |
| Yes | 482 (21.9) | 128 (38.1) |  |
| Missing |  | 3 (0.9) |  |
| Dementia and Parkinson’s disease (%) | 328 (14.2) | 61 (27.0) | <.001 |
| No | 1880 (85.4) | 249 (74.1) |  |
| Yes | 321 (14.6) | 83 (24.7) |  |
| Missing |  | 4 (1.2) |  |
| Depressive symptoms (%) |  |  |  |
| No | 1898 (86.2) | 264 (78.6) | .001 |
| Yes | 303 (13.8) | 72 (21.4) |  |
| ^a^ Numbers and percentages are provided unless otherwise specified.  ^b^ Calculated using the χ^2^ test with the exception for age (the analysis of variance). | | | |

| eTable 2. Baseline population characteristics by olfaction status (n=2348) ^a^ | | | | |
| --- | --- | --- | --- | --- |
| Characteristics | Olfaction status (B-SIT Score range) | | | |
|  | Anosmia (0-6)  (n = 328) | Hyposmia (7-8)  (n = 407) | Moderate (9-10)  (n = 809) | Good (11-12)  (n = 804) |
| Mean age (SD), year | 76.4 ± 2.9 | 75.8 ± 2.9 | 75.6 ± 2.9 | 75.1 ± 2.6 |
| Sex (%) |  |  |  |  |
| Men | 211 (64.3) | 219 (53.8) | 390 (48.2) | 307 (38.2) |
| Women | 117 (35.7) | 188 (46.2) | 419 (51.8) | 497 (61.8) |
| Race (%) |  |  |  |  |
| White | 156 (47.6) | 251 (61.7) | 506 (62.6) | 555 (69.0) |
| Black | 172 (52.4) | 156 (38.3) | 303 (37.4) | 249 (31.0) |
| Study site (%) |  |  |  |  |
| Memphis | 159 (48.5) | 212 (52.1) | 405 (50.1) | 351 (43.7) |
| Pittsburgh | 169 (51.5) | 195 (47.9) | 404 (49.9) | 453 (56.3) |
| Education (%) |  |  |  |  |
| Less than high school | 111 (33.8) | 112 (27.5) | 173 (21.4) | 118 (14.7) |
| High school | 93 (28.4) | 131 (32.2) | 279 (34.5) | 275 (34.2) |
| Above high school | 124 (37.8) | 164 (40.3) | 357 (44.1) | 411 (51.1) |
| Smoking (%) |  |  |  |  |
| Non-smoker | 132 (40.2) | 182 (44.7) | 353 (43.6) | 405 (50.4) |
| Ever-smoker | 196 (59.8) | 225 (55.3) | 456 (56.4) | 399 (49.6) |
| Drinking alcohol (%) |  |  |  |  |
| Never | 79 (24.1) | 118 (29.0) | 225 (27.8) | 233 (29.0) |
| Ever | 249 (75.9) | 289 (71.0) | 584 (72.2) | 571 (71.0) |
| Brisk walking (%) |  |  |  |  |
| <90 min/week | 302 (92.1) | 367 (90.2) | 731 (90.4) | 706 (87.8) |
| ≥90 min/week | 26 (7.9) | 40 (9.8) | 78 (9.6) | 98 (12.2) |
| Body mass index (kg/m^2^, %) |  |  |  |  |
| <25 | 117 (35.7) | 153 (37.6) | 255 (31.5) | 256 (31.8) |
| 25-30 | 143 (43.6) | 161 (39.6) | 340 (42.0) | 355 (44.2) |
| >30 | 68 (20.7) | 93 (22.8) | 214 (26.5) | 193 (24.0) |
| General health status (%) |  |  |  |  |
| Fair to poor | 79 (24.1) | 84 (20.6) | 118 (14.6) | 112 (13.9) |
| Excellent to good | 249 (75.9) | 323 (79.4) | 691 (85.4) | 692 (86.1) |
| Diabetes (%) | 91 (27.7) | 94 (23.1) | 192 (23.7) | 162 (20.2) |
| Cardiovascular disease (%) | 85 (25.9) | 121 (29.7) | 228 (28.2) | 221 (27.5) |
| Chronic kidney diseases (%) | 106 (32.3) | 103 (25.3) | 168 (20.8) | 156 (19.4) |
| Dementia and Parkinson’s disease (%) | 110 (33.5) | 82 (20.2) | 100 (12.4) | 62 (7.7) |
| Depressive symptoms (%) | 59 (18.0) | 65 (16.0) | 122 (15.1) | 93 (11.6) |
| Abbreviations: B-SIT, Brief Smell Identification Test  ^a^ Values are numbers (percentages) unless otherwise indicated. | | | | |

| eTable 3. Olfaction and grip strength, excluding prevalent cases of dementia or Parkinson’s disease | | | |
| --- | --- | --- | --- |
|  | Between-group difference with good olfaction as the reference (95% confidence interval) ^a^ | | |
|  | Men  N = 930 |  | Women  N= 1064 |
| Olfaction & baseline grip strength (kilograms) ^b^ | | | |
| Anosmia | -0.28 (-1.80, 1.19) |  | 0.02 (-1.23, 1.25) |
| Hyposmia | -2.01 (-3.47, -0.61) |  | 0.02 (-0.85, 0.98) |
| Moderate | -1.09 (-2.26, 0.09) |  | -0.59 (-1.28, 0.11) |
| Good | Ref. |  | Ref. |
| Annual decline in participants with good olfaction (kilograms/year) | -0.79 (-0.90, -0.69) |  | -0.42 (-0.48, -0.36) |
| Olfaction & annual decline in grip strength (kilograms) | | | |
| Anosmia | -0.27 (-0.46, -0.07) |  | -0.25 (-0.42, -0.08) |
| Hyposmia | 0.10 (-0.07, 0.29) |  | 0.00 (-0.13, 0.13) |
| Moderate | 0.01 (-0.13, 0.16) |  | -0.02 (-0.11, 0.08) |
| Good | Ref. |  | Ref. |
| ^a^ Estimated were obtained from joint models, adjusting for age, race, clinical site, education, smoking, alcohol drinking, brisk walking, body mass index, general health status, cardiovascular diseases, chronic kidney diseases, depressive syndromes, and dementia and Parkinson’s disease, and the loss of follow-up and death.  ^b^ Estimated between-group difference in grip strength at baseline, with good olfaction as the reference. | | | |

| eTable 4. Olfaction and quadriceps strength, excluding prevalent cases of dementia or Parkinson’s disease | | | |
| --- | --- | --- | --- |
|  | Between-group difference with good olfaction as the reference (95% confidence interval) ^a^ | | |
|  | Men  N = 883 |  | Women  N= 1139 |
| Olfaction & baseline quadriceps strength (Newton-meters) ^b^ | | | |
| Anosmia | -1.59 (-8.40, 5.22) |  | -6.35 (-11.6, -1.10) |
| Hyposmia | -4.92 (-11.2, 1.38) |  | -0.10 (-4.15, 3.95) |
| Moderate | -1.76 (-7.00, 3.48) |  | -2.07 (-5.06, 0.93) |
| Good | Ref. |  | Ref. |
| Annual decline in participants with good olfaction (Newton-meters/year) | -2.86 (-3.47, -2.24) |  | -1.98 (-2.29, -1.66) |
| Olfaction & annual decline in quadriceps strength (Newton-meters) | | | |
| Anosmia | -1.28 (-2.43, -0.14) |  | 1.39 (0.46, 2.32) |
| Hyposmia | -0.57 (-1.60, 0.46) |  | 0.15 (-0.52, 0.83) |
| Moderate | -0.78 (-1.62, 0.06) |  | 0.12 (-0.36, 0.60) |
| Good | Ref. |  | Ref. |
| ^a^ Estimated were obtained from joint models, adjusting for age, race, clinical site, education, smoking, alcohol drinking, brisk walking, body mass index, general health status, cardiovascular diseases, chronic kidney diseases, depressive syndromes, and dementia and Parkinson’s disease, and the loss of follow-up and death.  ^b^ Estimated between-group difference in quadriceps strength at baseline, with good olfaction as the reference. | | | |


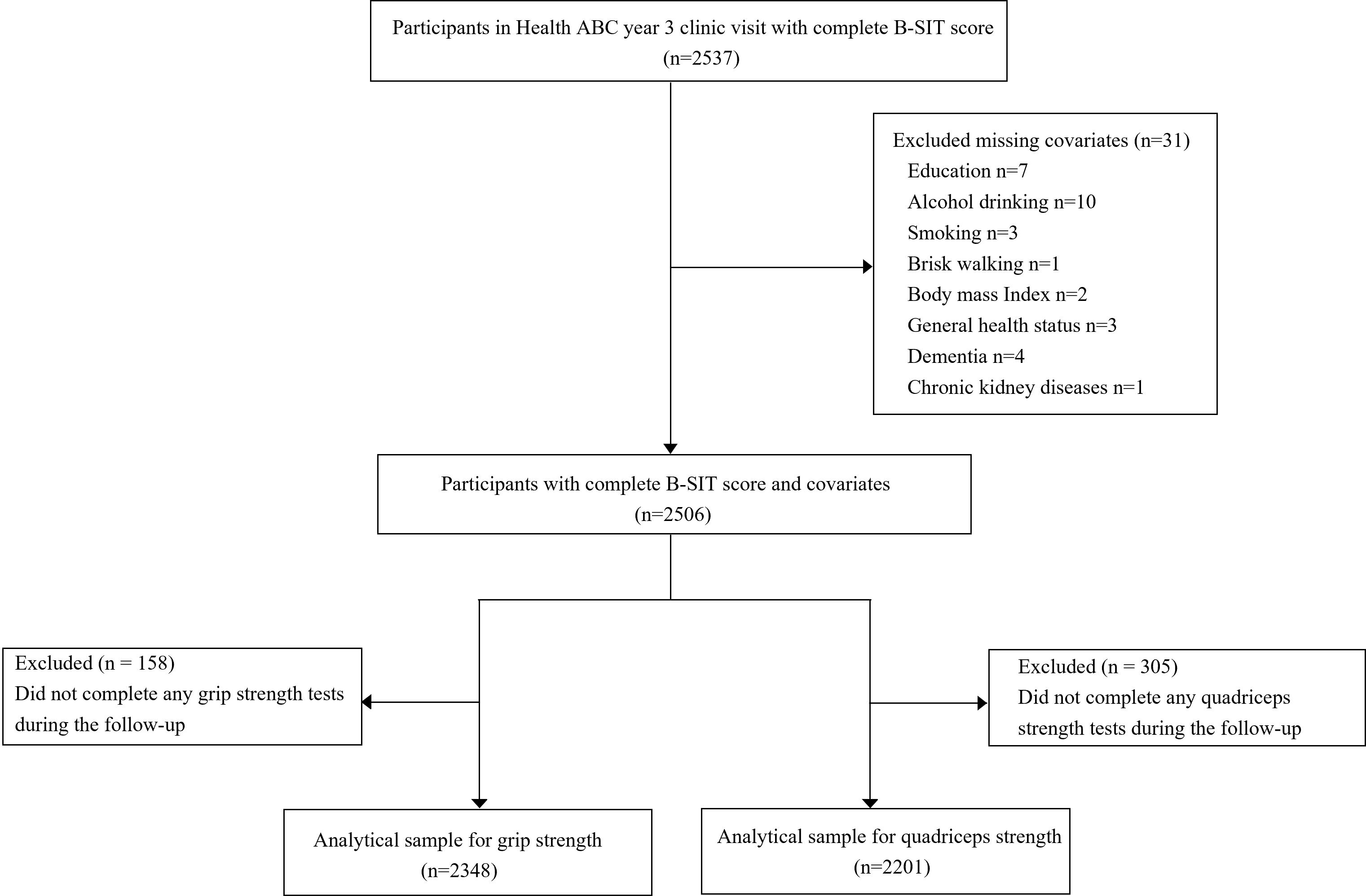


eFigure 1. Flowchart of study participation


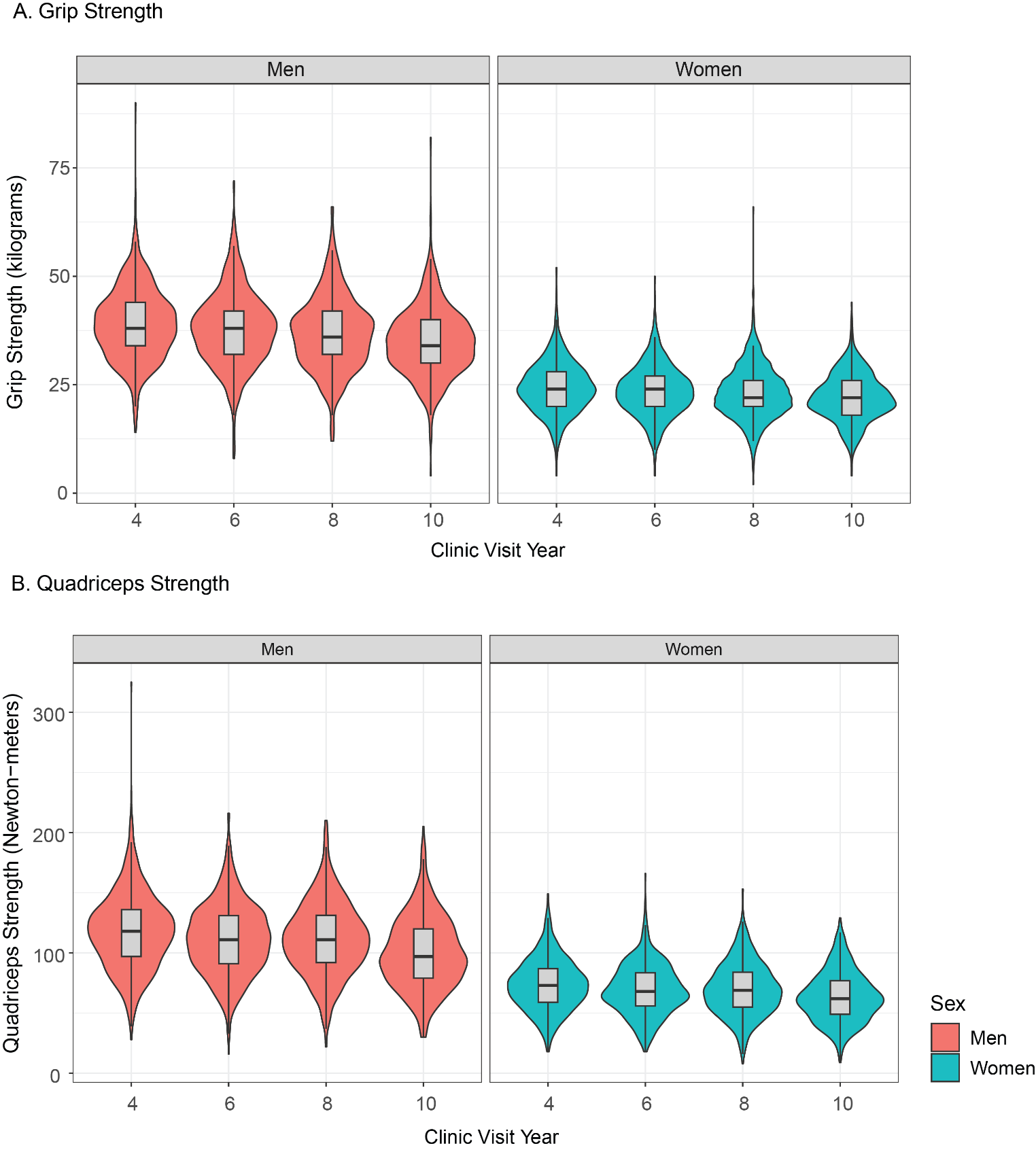


eFigure 2. Distribution of muscle strength, by clinic visit years and sex
